# Guanidinoacetic acid as a partial replacement to arginine with or without betaine in broilers offered moderately low crude protein diets

**DOI:** 10.1016/j.psj.2021.101692

**Published:** 2021-12-30

**Authors:** Nishchal K. Sharma, David J. Cadogan, Peter V. Chrystal, Peter McGilchrist, Stuart J. Wilkinson, Vivienne Inhuber, Amy F. Moss

**Affiliations:** ⁎ School of Environmental and Rural Science, University of New England, Armidale, New South Wales 2351, Australia; † Feedworks Pty Ltd, Romsey, Victoria 3434, Australia; ‡ Poultry Research Foundation, University of Sydney, Camden, New South Wales 2570, Australia; § AlzChem Trostberg GmbH, 83308, Trostberg, Germany

**Keywords:** arginine, betaine, broiler, guanidinoacetic acid, low crude protein diet

## Abstract

Guanidinoacetic acid (**GAA**) is the direct precursor of creatine and can spare arginine (**Arg**) for creatine synthesis in low crude protein (**CP**) broiler diets. This study aimed to determine the extent GAA could spare Arg in broilers offered low CP diets and if supplemental betaine provides additional benefits. Seven hundred twenty-day-old Ross 308 male broilers were assigned into 9 dietary treatments with 8 replicates of 10 birds each. The treatments were; normal CP diet, a low CP (−15 g/kg) diet deficient in Arg, a low CP diet sufficient in Arg, and low CP diets with GAA, where 0.1% added L-Arg was spared by GAA at 50, 100, and 150% with and without 0.1% betaine. The treatments were offered during grower (d 10–24) and finisher (d 25–42) phases. The birds offered a low CP Arg deficient diet had 7.8% lower weight gain, 10 points higher FCR, 8.5% lower breast meat yield, 27.2% lower breast meat creatine concentration and 30.4% more abdominal fat pad compared to those offered a normal CP diet. When Arg was added back to the Arg deficient diet, growth performance, breast meat yield and creatine concentration loss were restored. When GAA spared Arg at 150%, feed intake, weight gain, FCR, breast and abdominal fat yields, breast meat moisture, drip loss, and breast meat creatine concentration became comparable to Arg sufficient low CP and normal CP treatments. When GAA spared Arg at 100 and 50%, FCR was 3 and 5 points lower than the normal CP treatment. Breast meat creatine concentration was positively correlated to feed efficiency (r = 0.70, *P* < 0.001) and breast meat moisture (r = 0.33, *P* < 0.01), and negatively correlated to relative weight of abdominal fat (r = −0.37, *P* < 0.01) and breast meat pH (r = −0.49, *P* < 0.001). There were no benefits of adding betaine with GAA on the parameters measured but the results with GAA were consistent in the presence or absence of betaine.

## INTRODUCTION

Creatine (**Cr**) plays a vital role in energy metabolism and the formation of muscles and other tissues in the body (Brosnan et al., 2009). A 3-wk-old broiler of 985 g average body weight requires 169 mg of Cr daily (Khajali et al., 2020) and this requirement increases with age. Only two-thirds of the daily Cr requirements may be synthesized endogenously and the rest should be supplemented through feed (Tossenberger et al., 2016). Animal derived feed ingredients such as meat and bone meal and fish meal are rich sources of Cr (Wyss and Kaddurah-Daouk, 2000) but processing and heat treatment significantly diminish their Cr contents (Tossenberger et al., 2016) resulting in big variation in Cr contents between batches. Vegetable protein sources and grains, on the other hand, have negligible concentrations of Cr (Khajali et al., 2020). Thus, dietary Cr or its precursors are naturally low in low crude protein (**CP**) broiler diets as less protein meals and more crystalline amino acids are used. This puts an additional burden on *de novo* Cr synthesis, which is not adequate to meet the bird's requirement for Cr, and thus growth performance of broilers is compromised.

Creatine is synthesized endogenously from the amino acids arginine (**Arg**) and glycine (**Gly**). When Gly receives a guanidine group from Arg in a reaction catalyzed by the enzyme L-Arg:Gly amidinotransferase (**AGAT**) mainly in the kidney, guanidinoacetic acid (**GAA**) is produced. GAA is then transported to the liver where it is methylated by S-adenosyl methionine (**SAM**) in a reaction catalyzed by the enzyme guanidinoacetate N-methyltransferase to form Cr. This Cr is transported from the liver to the cells with high energetic requirements such as skeletal muscles, heart and brain, and gets phosphorylated to form phosphocreatine. Phosphocreatine converts adenosine diphosphate (**ADP**) into adenosine triphosphate (**ATP**) during its dephosphorylation (Khajali et al., 2020; Portocarero and Braun, 2021). Thus, cellular Cr serves as an energy storage molecule and can produce ATP on demand for energy supply (Wyss and Kaddurah-Daouk, 2000). This is particularly important during periods of high energy demand, such as rapid muscle growth in broilers, and prevents the formation of reactive oxygen substances which negatively affect performance. As described above, two amino acids Arg and Gly are the precursors for endogenous Cr synthesis both of which are limiting in low CP diets. The formation of GAA from Arg and Gly is regulated by a negative feedback mechanism that involves circulating Cr and ornithine concentrations on AGAT activity (Wyss and Kaddurah-Daouk, 2000). Thus, Cr synthesis and its concentration in muscle tissues may not be increased beyond such regulatory levels by simply supplying Arg or Gly in low CP diets. Dietary GAA supplementation would bypass this rate regulating mechanism to increase Cr synthesis in the body (Khajali et al., 2020).

Creatine is not an effective feed additive for poultry because it is unstable and costly Baker, 2009. GAA is a direct precursor of Cr and is stable in aqueous solution and pellet feed processing (Vranes et al., 2017; Van der Poel et al., 2018). GAA as a feed additive has shown benefits on growth performance and breast meat yield of broilers when it was added either on top of a complete feed or as a replacement to Arg in feed (Tossenberger et al., 2016; DeGroot et al., 2018, 2019). Theoretically, GAA has an Arg sparing potential of up to 149% in broilers (Khajali et al., 2020) but a more conservative approach of using a 77% replacement rate without exceeding 0.12% inclusion rates is common in the poultry industry. While dietary supplementation of L-Arg has less effect on energy metabolism and muscle Cr concentrations, dietary supplementation of GAA has shown to improve muscle Cr level, phosphocreatine to ATP ratio in muscles, and increased serum Arg level (DeGroot et al., 2018, 2019). Dietary supplementation of GAA has also been shown to increase breast meat yield (Michiels et al., 2012) and reduce wooden breast severity in broilers (Córdova-Noboa et al., 2018). The Arg sparing potential of GAA would be of considerable interest in low CP diets as they have lower inclusions of protein meals, lower concentrations of Cr, and a significant amount of supplemental L-Arg which may be cleared quickly in the blood and not an efficient way of maximizing muscle Cr formation and growth promotion.

A recent study (Dao et al., 2021) showed that the response of broilers to supplemental GAA in Arg deficient low CP diet was minimal. This was possibly due to excessive dietary CP reduction, a high level of GAA supplementation, and insufficient methyl donors to convert GAA to Cr. The methyl group donated by SAM to GAA originates from methionine. The hepatic synthesis of Cr from GAA uses a significant proportion of methyl group and this can limit methionine available for protein synthesis. Adding in more methionine as a methyl donor will increase the potentially toxic homocysteine, therefore, betaine (trimethylglycine) may be a better supplement as betaine converts homocysteine back to methionine, and once it has donated its methyl group, it gets converted to Gly. Betaine may thereby restore balance in metabolism, stopping the build-up of potentially toxic homocysteine, and sparing methionine and Gly (Ostogic et al., 2013; Maidin et al., 2021). Thus, betaine in combination with GAA may be useful in low CP diets for broilers. Therefore, this study was conducted to determine the extent GAA may spare Arg in broilers offered moderately low CP diets and if supplemental betaine provides additional benefits on growth performance, carcass yield, and meat quality.

## MATERIALS AND METHODS

### Animal Ethics

This experiment was approved by the animal ethics committee of the University of New England, Australia (Authority No. AEC21-005). All broiler management procedures including health care, husbandry and use of laboratory animals fulfilled the requirements of the Australian Code for the Care and Use of Animals for Scientific Purposes (NHMRC, 2013).

### Experimental Design and Bird Management

A total of 720-day-old Ross 308 off-sex male broiler chicks were sourced from Baiada hatchery in Goulburn, New South Wales. On d 0, chicks were randomly assigned to 72 floor pens of equal size in an environmentally controlled poultry research facility. Each pen was equipped with a feeder and 2 nipple drinkers. The birds had ad libitum access to feed and water throughout the study. The pens were spread with hard wood shavings up to a depth of approximately 7 cm. The lighting and temperature followed the Ross 308 breed guidelines (Aviagen, 2018). On d 10, all birds were weighed and re-assigned to pens of approximately equal weight within 3% of the experiment mean for body weight and checked for no significant differences in weight between treatments. The birds were assigned into nine treatments with 8 replicates of 10 birds each. The treatments were as follows: a normal CP diet, a low CP diet (CP reduced by 15 g/kg) deficient in Arg (low CP − Arg), a low CP diet sufficient in Arg (low CP + Arg) and low CP diets with GAA (Creamino®, AlzChem, Germany) where 0.1% added L-Arg was spared by GAA at 50% (GAA inclusion rate- 0.2%), 100% (GAA inclusion rate- 0.1%) and 150% (GAA inclusion rate- 0.067%) with and without 0.1% betaine.

### Diet

The diets were thoroughly mixed and pelleted at a temperature of 65⁰ C at the University of New England, Australia. The basal diet contained wheat, sorghum, and soybean meal as major ingredients. The ingredients were analyzed for CP, metabolizable energy, total and digestible amino acids before diet formulation by using near-infrared spectroscopy (Foss NIR 6500, Denmark), standardized with Adisseo PNE advanced calibration.

The ingredient and calculated nutrient composition of the diets are presented in Tables 1 and 2, respectively. A standard starter crumble (2,975 kcal/kg AMEn, 23.3% CP) was offered for the first 10 d post-hatch. The treatment grower and finisher diets were offered as 3-mm pellets during d 10 to 24 and d 24 to 42, respectively. The diets were formulated to meet the Ross 308 nutrient specifications (Aviagen, 2019a) and the ratio of d Arg to d Lysine (**Lys**) was set at 105% as per commercial practice. The CP contributions from supplemental AA were included in diet formulations. The normal CP diet was balanced for digestible AA by adding supplemental L-lysine, D,L-methionine, and L-threonine. The low CP diet contained 15 g/kg lower CP than the normal CP diet. The digestible AA in a low CP diet was balanced by adding L-valine, L-Arg, L-isoleucine, and L-Gly in addition to L-Lys, D,L-methionine and L-threonine. Diets were supplemented with xylanase (Axtra XB 201 TPT, Dupont Animal Nutrition, UK) and phytase (Quantam Blue 5G, AB Vista Feed Ingredient, UK). Vitamin and mineral premixes were added to meet requirements following manufacturer recommendations (UNE VM, Rabar Pty Ltd., Australia; UNE TM, Rabar Pty Ltd., Australia).

**Table 1:** Ingredient composition of the experimental diets.

Ingredients, %	Starter diet	Grower diet			Finisher diet		
		Normal CP^1^	Low CP - Arg^2^	low CP + Arg^3^	Normal CP	Low CP - Arg	low CP + Arg
Wheat (11.4% CP)	32.18	36.65	43.88	43.88	41.13	48.26	48.26
Sorghum (10.5% CP)	25.00	25.00	25.00	25.00	25.00	25.00	25.00
Soybean meal (46.5% CP)	35.14	30.46	23.15	23.15	25.50	18.25	18.25
Canola oil	3.27	4.35	3.28	3.28	5.23	4.19	4.19
Limestone	1.064	0.831	0.805	0.805	0.674	0.648	0.648
Dicalcium phosphate	1.776	1.248	1.391	1.391	1.055	1.197	1.197
Sodium chloride	0.120	0.124	0.047	0.047	0.125	0	0
Sodium bicarbonate	0.356	0.352	0.464	0.464	0.351	0.534	0.534
Potassium carbonate	0	0	0	0	0	0.016	0.016
Choline chloride, 70%	0.032	0.054	0.089	0.089	0.079	0.113	0.113
L-lysine HCl	0.302	0.275	0.490	0.490	0.257	0.471	0.471
D,L-methionine	0.352	0.307	0.362	0.362	0.275	0.329	0.329
L-threonine	0.153	0.124	0.220	0.220	0.099	0.195	0.195
L-valine	0.023	0.005	0.120	0.120	0	0.108	0.108
L-arginine	0.009	0	0	0.200	0	0	0.198
Guanidinoacetic acid^4^	0	0	0	0	0	0	0
L-isoleucine	0	0	0.098	0.098	0	0.096	0.096
L-glycine	0	0	0.179	0.179	0	0.178	0.178
Xylanase^5^	0.010	0.010	0.010	0.010	0.010	0.010	0.010
Phytase^6^	0.010	0.010	0.010	0.010	0.010	0.010	0.010
Vitamin premix^7^	0.085	0.080	0.080	0.080	0.080	0.080	0.080
Mineral premix^8^	0.110	0.100	0.100	0.100	0.100	0.100	0.100

1 Diet 1- Normal crude protein diet.

2 Diet 2- Low crude protein diet deficient in Arginine.

3 Diet 3- Low crude protein diet sufficient in Arginine.

4 Diets 4 to 9- low CP diets with GAA where 0.1% added L-Arg was spared by GAA at 50% (inclusion rate- 0.2%), 100% (inclusion rate- 0.1%), and 150% (inclusion rate- 0.067%) with and without 0.1 % betaine.

5 Axtra XB 201 TPT, Dupont Animal Nutrition, UK.

6 Quantam Blue 5G, AB Vista Feed Ingredient, UK.

7 Vitamin premix per kg diet (UNE VM, Rabar Pty Ltd): vitamin A, 12 MIU; vitamin D, 5 MIU; vitamin E, 75 mg; vitamin K, 3 mg; nicotinic acid, 55 mg; pantothenic acid, 13 mg; folic acid, 2 mg; riboflavin, 8 mg; cyanocobalamin, 0.016 mg; biotin, 0.25 mg; pyridoxine, 5 mg; thiamine, 3 mg; antioxidant, 50 mg.

8 Mineral premix per kg diet (UNE TM, Rabar Pty Ltd): Cu, 16 mg as copper sulfate; Mn, 60 mg as manganese sulfate; Mn, 60 mg as manganous oxide; I, 0.125 mg as potassium iodide; Se, 0.3 mg; Fe, 40 mg, as iron sulfate; Zn, 50 mg as zinc oxide; Zn, 50 mg as zinc sulfate.

**Table 2:** Calculated nutrient composition of the experimental diets (as-fed basis).

Nutrient composition, %	Starter diet	Grower diet			Finisher diet		
		Normal CP^1^	Low CP - Arg^2^	low CP + Arg^3^	Normal CP	Low CP - Arg	low CP + Arg
AMEn, kcal/kg	2,975	3,100	3,100	3,100	3,200	3,200	3,200
CP	23.3	21.5	20.0	20.0	19.7	18.2	18.2
Crude fat	5.44	6.51	5.48	5.48	7.39	6.38	6.38
Crude fibre	2.41	2.36	2.29	2.29	2.30	2.24	2.24
Guanidinoacetic acid^4^	0	0	0	0	0	0	0
Dig Arg: Dig Lys	1.050	1.050	0.877	1.050	1.050	0.856	1.050
Dig Lys^5^	1.280	1.150	1.150	1.150	1.020	1.020	1.020
Dig Met	0.641	0.577	0.600	0.600	0.524	0.547	0.547
Dig M+C	0.950	0.870	0.870	0.870	0.800	0.800	0.800
Dig Thr	0.860	0.770	0.770	0.770	0.680	0.680	0.680
Dig Val	0.960	0.870	0.870	0.870	0.786	0.780	0.780
Dig Arg	1.344	1.208	1.008	1.208	1.071	0.873	1.071
Dig Ile	0.869	0.796	0.780	0.780	0.718	0.700	0.700
Dig Trp	0.264	0.243	0.211	0.211	0.221	0.188	0.188
Dig Gly_eq_^6^	1.459	1.345	1.345	1.345	1.222	1.222	1.222
Calcium	1.09	0.87	0.87	0.87	0.75	0.75	0.75
Available P	0.52	0.44	0.44	0.44	0.39	0.39	0.39
Sodium	0.18	0.18	0.18	0.18	0.18	0.18	0.18
Chloride	0.22	0.22	0.22	0.22	0.22	0.19	0.19

1 Diet 1- Normal crude protein diet.

2 Diet 2- Low crude protein diet deficient in Arginine.

3 Diet 3- Low crude protein diet sufficient in Arginine.

4 Diets 4 to 9- low CP diets with GAA where 0.1% added L-Arg was spared by GAA at 50% (GAA inclusion rate- 0.2%), 100% (GAA inclusion rate- 0.1%), and 150% (GAA inclusion rate- 0.067%) with and without 0.1% betaine.

5 Digestible coefficients of AA for raw materials were determined using AMINODat 5.0 (Evonik Animal Nutrition).

6 Digestible Gly_eq_ was calculated as follows: Dig Gly_eq_= Dig Gly + (Dig Ser × 0.7143).

Dry matter contents of finished feeds were determined by subjecting samples to a forced air oven at 105°C until constant weight. Nitrogen contents of feeds were determined on a 0.25 g sample with a combustion analyzer (Leco model FP-2000 N analyzer, Leco Corp., St. Joseph, MI) using EDTA as a calibration standard, with CP being calculated by multiplying percentage Nitrogen by a correction factor (6.25). Amino acid profiles of feeds were determined at the Australian Proteome Analysis Facility, Macquarie University, Sydney. Gross energy contents of feeds were determined on a 0.5 g sample using an adiabatic bomb calorimeter (IKA Werke, C7000, GMBH and Co., Staufen, Germany) with benzoic acid as standard.

### Growth Performance

Birds and feed were weighed on arrival and d 10, 24, and 42, and mortality was recorded daily. Feed intake, weight gain, and mortality adjusted feed conversion ratio (**FCR**) were determined during the experimental periods.

### Carcass Cuts and Internal Organs

On d 42, four birds were sampled per pen, weighed and euthanized by an electrical stunner (MEFE CAT 44N, Mitchell Engineering Food Equipment, Clontarf, QLD, Australia) followed by cervical dislocation. Breast meat, leg piece (thigh and drumstick), liver, and abdominal fat pad were collected. The relative weights of carcass cuts and internal organs were calculated as mass per unit of live body weight (g/kg of live body weight). The left toe was collected to measure toe ash percentage.

### Creatine and GAA Concentrations in Tissues and Finished Feeds

Around 400 g of feed was collected from each treatment and shipped to AlzChem Trostberg GmbH lab in Germany to measure GAA concentrations. On d 42, four birds were sampled per pen (32 birds/treatment) to measure Cr concentration in breast meat. For Cr measurement, around 80 g of breast meat was collected from the cranial portion of the right breast muscle and packed individually in freezer bags. The samples were stored frozen at −20° C and shipped on dry ice to AlzChem Trostberg GmbH lab in Germany.

### Meat Quality

Meat quality parameters were measured on breast meat samples collected from four birds per pen (32 birds per treatment) by the following procedures.

### White Striping and Wooden Breast

The right breast meat was visually assessed for the presence or absence of woody breast according to the method described by Kuttappan et al. (2016). The data was recorded as the percentage of birds with wooden breasts. The white stripping severity was recorded on a 4-point scale following the procedures of Bailey et al. (2015). In short, score 0 represented no white striping, score 1 was mild noticeable striping covering part of the breast, score 2 was moderate noticeable striping covering the breast surface extensively and score 3 was severe and very thick striping covering the breast surface extensively.

### Drip Loss

The left breast meat was excised with bone, weighed, and placed into individually labeled Ziploc bags. Then the samples were immediately stored at 4°C in a fridge hanging in Ziploc bags. At 24 h postmortem, the breast meat samples were re-weighed to calculate drip loss by using the following equation.

Drip loss={day1breastweight(g)−day 2breast weight (g)}/day 1breast weight (g)*100

After drip loss measurement, the breast meat was removed from the bone and cut into 2 portions. The cranial half portion was used for measuring color and pH. The caudal half portion was used for measuring moisture, cooking loss, and shear force.

### Moisture

Around 60 g of breast meat samples were freeze-dried at −50⁰C until constant weight to calculate the moisture content of the sample by using the following equation.

Breast meat moisture,%={Moist breast weight (g)−dry breast weight (g)}/Moist breast weight×100

### pH and Color

A hand-held pH meter (IJ44C probe, Ionode Pty Ltd., Tennyson, QLD, Australia) with an integrated temperature sensor (WP-80 Waterproof pH-mV-Temperature Meter, TPS, Brendale, NSW, Australia) was used to measure the pH of a cranial half portion of left breast meat at 24-h postmortem. The pH meter was calibrated at room temperature using buffers at pH 4.0 and 6.9 and the measurements were made in duplicates by inserting the pH probe at approximately 2.5 cm from the top of the breast. After measuring pH, breast meat color (lightness- L*, redness- a*, and yellowness- b*) was measured in triplicates using a Minolta Chroma Meter CR-300 (Minolta Co., Ltd., Osaka, Japan). The instrument was calibrated using a white tile (Y = 93.3, x = 0.3135, y = 0.3198; Minolta Co., Ltd., Osaka, Japan) using illuminant D-65. Three readings were taken on each sample and averaged. Values of lightness (L*), redness (a*), and yellowness (b*) were recorded.

### Cooking Loss and Shear Force

Around 65 g of breast meat samples were individually vacuum packed in vacuum pouches and stored at −20° C until cooking loss and shear force were determined. The cooking loss and shear force measurements were performed based on the methods described by Hopkins et al. (2010). For cooking loss, the frozen samples were placed into a water bath (Model: BTC 9090, Thermoline, Sydney, NSW, Australia) set at 85° C for 25 min and then placed under cold running tap water for 30 min to stop the cooking process. Samples were then removed from the bag, wrapped in paper towels to remove the excess water, and weighed (cooked sample weight) for cooking loss determination. After cooking loss measurements, the samples were stored in individual zip lock bags at 4°C overnight for subsequent shear force analysis. Shear force analysis was performed using a Lloyd Instruments LRX Materials Testing Machine fitted with a 500 N load cell (Lloyd Instruments Ltd., Hampshire, UK). Briefly, 5 subsamples with a rectangular cross-section of 15 mm width and 6.66 mm depth (1 cm^2^) were cut from each block, with fiber orientation parallel to the long axis right angles to the shearing surface. The force required to shear through the clamped subsample with a triangulated 0.64-mm thick blade pulled upward at a speed of 100 mm/min at a 90° angle to the fiber direction and was expressed as kg peak force. Values of the kg peak force were recorded, and the mean value obtained from the subsamples was converted to Newton values (N) (1 kg = 9.81 N) for statistical analysis.

### Statistical Analysis

The data were analyzed by one-way ANOVA using JMP statistical software version 14 (SAS Institute Inc, Cary, NC). When one-way ANOVA showed significant (*P* < 0.05) differences among treatments, treatment means were compared using the post hoc Tukey test. The wooden breast severity data were not normally distributed and the non-parametric Kruskal-Wallis test was used to detect the significance. Pearson correlation coefficients and associated significance were generated using JMP software to determine the relationship between breast meat Cr concentration and breast meat moisture, pH, abdominal fat, and FCR. The relationship between breast meat Cr concentration, FCR and abdominal fat pad was investigated by linear regression.

## RESULTS

The analyzed nutrient composition of starter, grower, and finisher diets are presented in Tables 3 and 4. The analyzed GAA contents in the diets were close to the calculated inclusion rates. On average, the analyzed GAA concentrations in grower and finisher diets were lower than the calculated values by 6.4 and 3.9%, respectively which suggests that Creamino® provided the desired levels of GAA in the diets considering a minimum of 96% GAA concentration in Creamino®. The analyzed Cr concentrations in the diets were below the minimum reporting limit set in the assay (i.e., 20 mg/kg) and thus not reported.

**Table 3:** Analyzed nutrient composition of starter and grower diets (as-fed basis).

Nutrient composition, %	Starter diet	Grower diets								
		Normal CP^1^	Low CP - Arg^2^	low CP + Arg^3^	Low CP + GAA 50^4^	Low CP + GAA 100^5^	Low CP + GAA 150^6^	Low CP + GAA 50 + Betaine^7^	Low CP + GAA 100 + Betaine^8^	Low CP + GAA 150 + Betaine^9^
DM^10^	88.2	87.5	87.2	87.0	87.1	87.0	87.0	87.1	87.3	87.5
GE^11^, kcal/kg	4063	4111	4049	4025	4028	4015	4018	4030	4032	4044
CP^12^	24.5	22.6	20.4	20.7	21.0	20.8	20.6	20.6	21.0	20.5
GAA^13^	< RL	< RL	< RL	< RL	0.165	0.101	0.072	0.179	0.104	0.064
Lys	1.42	1.28	1.28	1.23	1.23	1.24	1.25	1.25	1.27	1.24
Met	0.63	0.48	0.54	0.53	0.54	0.53	0.52	0.52	0.55	0.55
Thr	1.00	0.90	0.87	0.87	0.86	0.86	0.88	0.88	0.88	0.86
Val	1.12	1.04	1.01	1.00	1.00	0.98	1.02	1.01	1.02	0.99
Arg	1.46	1.32	1.09	1.27	1.19	1.16	1.21	1.22	1.17	1.17
Ile	1.02	0.96	0.91	0.90	0.89	0.88	0.91	0.91	0.89	0.88
Leu	1.88	1.79	1.56	1.55	1.54	1.50	1.57	1.57	1.52	1.54
Gly	0.96	0.89	0.95	0.94	0.93	0.92	0.95	0.95	0.97	0.94
Ser	1.15	1.07	0.92	0.92	0.92	0.89	0.93	0.93	0.90	0.91
Phe	1.17	1.10	0.95	0.95	0.94	0.92	0.96	0.96	0.93	0.92
His	0.59	0.55	0.47	0.47	0.46	0.46	0.48	0.47	0.46	0.47
Tyr	0.63	0.60	0.48	0.50	0.50	0.48	0.51	0.53	0.47	0.49
Pro	1.40	1.36	1.25	1.26	1.25	1.23	1.27	1.27	1.24	1.28
Ala	1.06	1.01	0.90	0.89	0.89	0.86	0.90	0.90	0.87	0.89
Asp	2.29	2.09	1.75	1.74	1.73	1.68	1.77	1.76	1.71	1.69
Glu	4.77	4.57	4.17	4.19	4.15	4.08	4.24	4.23	4.13	4.07

1 Diet 1- Normal crude protein diet.

2 Diet 2- Low crude protein diet deficient in Arginine.

3 Diet 3- Low crude protein diet sufficient in Arginine.

4-9 Diets 4 to 9- low CP diets with GAA where 0.1% added L-Arg was spared by GAA at 50% (GAA inclusion rate- 0.2%), 100% (GAA inclusion rate- 0.1%), and 150% (GAA inclusion rate- 0.067%) with and without 0.1% betaine.

10 Dry matter.

11 Gross energy.

12 Crude protein.

13 Guanidinoacetic acid levels in the diets, RL denotes the minimum reporting limit which was set at 20 mg/kg.

**Table 4:** Analyzed nutrient composition of finisher diets (as-fed basis).

Nutrient composition, %	Finisher diet								
	Normal CP^1^	Low CP - Arg^2^	low CP + Arg^3^	Low CP + GAA 50^4^	Low CP + GAA 100^5^	Low CP + GAA 150^6^	Low CP + GAA 50 + Betaine^7^	Low CP + GAA 100 + Betaine^8^	Low CP + GAA 150 + Betaine^9^
DM^10^	87.0	87.0	87.0	87.0	87.5	87.0	87.1	87.2	87.4
GE^11^, kcal/kg	4125	4075	4089	4071	4077	4063	4065	4084	4085
CP^12^	20.2	18.7	19.1	19.3	18.9	18.6	18.4	19.0	18.9
GAA^13^	< RL	< RL	< RL	0.183	0.098	0.067	0.192	0.099	0.066
Lys	1.10	1.11	1.10	1.10	1.10	1.07	1.09	1.09	1.06
Met	0.42	0.49	0.49	0.46	0.49	0.48	0.49	0.52	0.45
Thr	0.78	0.79	0.79	0.79	0.78	0.74	0.75	0.76	0.76
Val	0.91	0.96	0.93	0.94	0.95	0.88	0.88	0.89	0.88
Arg	1.11	0.99	1.16	1.06	1.06	1.00	0.99	1.04	1.02
Ile	0.83	0.85	0.83	0.85	0.83	0.78	0.78	0.79	0.78
Leu	1.56	1.48	1.46	1.49	1.48	1.39	1.34	1.38	1.34
Gly	0.79	0.91	0.89	0.89	0.88	0.84	0.85	0.87	0.83
Ser	0.93	0.88	0.87	0.87	0.87	0.80	0.78	0.81	0.80
Phe	0.95	0.90	0.90	0.91	0.90	0.82	0.81	0.83	0.82
His	0.48	0.45	0.45	0.45	0.45	0.42	0.41	0.42	0.42
Tyr	0.46	0.44	0.44	0.42	0.44	0.42	0.40	0.44	0.41
Pro	1.28	1.36	1.36	1.36	1.36	1.18	1.16	1.19	1.19
Ala	0.89	0.83	0.82	0.84	0.84	0.80	0.77	0.80	0.77
Asp	1.74	1.49	1.47	1.49	1.48	1.42	1.38	1.42	1.40
Glu	4.11	4.24	4.24	4.23	4.23	3.69	3.66	3.71	3.75

1 Diet 1- Normal crude protein diet.

2 Diet 2- Low crude protein diet deficient in Arginine.

3 Diet 3- Low crude protein diet sufficient in Arginine.

4-9 Diets 4 to 9- low CP diets with GAA where 0.1% added L-Arg was spared by GAA at 50% (GAA inclusion rate- 0.2%), 100% (GAA inclusion rate- 0.1%), and 150% (GAA inclusion rate- 0.067%) with and without 0.1% betaine.

10 Dry matter.

11 Gross energy.

12 Crude protein.

13 Guanidinoacetic acid levels in the diets, RL denotes the minimum reporting limit which was set at 20 mg/kg.

### Growth Performance

The overall mortality in this experiment during the experimental period of d 10 to 42 was 2.85 % and was not related to dietary treatment (*P* > 0.05). The performance of birds exceeded the Ross 308 performance standards (Aviagen, 2019b) for feed intake, weight gain and FCR (Table 5). During d 10 to 42, the average feed intake, weight gain, and FCR of birds offered a normal CP diet were 4,843 g, 3,194 g, and 1.517, respectively. Compared to the Aviagen performance standard for Ross 308, feed intake was higher by 2.98% (4,843 g vs. 4,703 g), weight gain was higher by 13.5% (3,194 g vs. 2,813 g), and FCR was lower by 15 points (1.517 vs. 1.672) during d 10 to 42.

**Table 5:** Growth performance of broilers offered low crude protein diets with guanidinoacetic acid and betaine.

Treatment	Feed intake (g/bird)			Weight gain (g/bird)			FCR (g/g)		
	d 10–24	d 24–42	d 10–42	d 10–24	d 24–42	d 10–42	d 10–24	d 24–42	d 10–42
Normal CP^1^	1,499^a^	3,344	4,843^a^	1,140^a^	2,053^a^	3,194^a^	1.315^bc^	1.629^b^	1.517^bc^
Low CP - Arg^2^	1,460^b^	3,290	4,749abc	1,052^c^	1,894^b^	2,946^c^	1.388^a^	1.737^a^	1.612^a^
Low CP + Arg^3^	1,465^ab^	3,279	4,744abc	1,095^b^	2,047^a^	3,142^ab^	1.337^b^	1.602^bc^	1.510bcd
Low CP + GAA 50^4^	1,434^bc^	3,216	4,650^c^	1,103^b^	2,076^a^	3,179^ab^	1.301^c^	1.549^d^	1.463^e^
Low CP + GAA 100^5^	1,470^ab^	3,265	4,735abc	1,117^ab^	2,067^a^	3,184^ab^	1.316^bc^	1.581^cd^	1.488^d^
Low CP + GAA 150^6^	1,469^ab^	3,311	4,781^ab^	1100^b^	2,032^a^	3,132^ab^	1.337^b^	1.631^b^	1.527^b^
Low CP + GAA 50 + betaine^7^	1,450^bc^	3,213	4,663^bc^	1,107^b^	2,021^a^	3,129^ab^	1.310^bc^	1.590^c^	1.491^d^
Low CP + GAA 100 + betaine^8^	1,421^c^	3,235	4,645^c^	1,095^b^	2,012^a^	3,099^b^	1.297^c^	1.608^bc^	1.499^cd^
Low CP + GAA 150 + betaine^9^	1,447^bc^	3,241	4,689^bc^	1,090^b^	2,019^a^	3,109^ab^	1.328^b^	1.605^bc^	1.508bcd
SEM	4.47	11.29	14.14	4.04	9.51	12.07	0.004	0.007	0.005
*P* value	< 0.01	0.076	< 0.01	< 0.001	< 0.001	< 0.001	< 0.001	< 0.001	< 0.001

a-d Within each treatment factor, means in the same column with a different superscript differ significantly (*P* < 0.05).

1 Diet 1- Normal crude protein diet.

2 Diet 2- Low crude protein diet deficient in Arginine.

3 Diet 3- Low crude protein diet sufficient in Arginine.

4-9 Diets 4 to 9- low CP diets with GAA where 0.1% added L-Arg was spared by GAA at 50% (GAA inclusion rate- 0.2%), 100% (GAA inclusion rate- 0.1%) and 150% (GAA inclusion rate- 0.067%) with and without 0.1% betaine.

The effect of low CP diets with GAA and betaine on the growth performance of broilers is presented in Table 5. Dietary treatments had a significant effect on feed intake (*P* < 0.01) during d 10 to 24 and d 10 to 42. During d 10 to 24, the birds offered a low CP diet deficient in Arg had 2.60% lower feed intake as compared to those offered a normal CP diet. When Arg was added back, feed intake improved and became statistically similar to the normal CP treatment. When GAA spared Arg at 50, 100, and 150% without or with betaine, there was no effect on feed intake except that the birds offered a low CP diet with GAA 100 + betaine had lower feed intake compared to those offered a low CP diet with Arg or low CP diet with GAA 100. During d 10 to 42, the birds offered a low CP diet deficient in Arg or a low CP diet sufficient in Arg had a similar feed intake to those offered a normal CP diet. When GAA spared Arg at 50, 100, and 150% without or with betaine, there was no effect on feed intake compared to the low CP + Arg treatment. Betaine did not affect feed intake when it was added to each level of GAA. When GAA spared Arg at 50%, feed intake was lower compared to when GAA spared Arg at 150% and normal CP treatment. Dietary treatments tended (*P* = 0.076) to affect feed intake during d 24 to 42 and the highest feed intake during this period was observed in the birds offered a normal CP diet.

Dietary treatments had a significant effect on weight gain (*P* < 0.001) in all the phases. The birds offered a low CP diet deficient in Arg had around 7.8% lower weight gain as compared to those offered a normal CP diet in all the phases. When Arg was added back, weight gain increased in all the phases and became comparable to the normal CP treatment during d 24 to 42 and d 10 to 42. When GAA spared Arg at 50, 100, and 150% without or with betaine, weight gain was higher than the Arg deficient low CP treatment and comparable to the low CP + Arg treatment in all the phases. Betaine did not affect weight gain when it was added to each level of GAA.

Dietary treatments led to a significant difference in FCR (*P* < 0.001) in all the phases. The birds offered a low CP diet deficient in Arg had 7 points higher FCR in the grower phase and 10 points higher FCR in the finisher and overall phases as compared to those offered a normal CP diet. When Arg was added back, FCR decreased and became comparable to the normal CP treatment in all the phases. When GAA spared Arg at 100 and 150%, FCR was lower than the low CP – Arg treatment but comparable to the low CP + Arg treatment in all the phases. When GAA spared Arg at 50%, FCR was lower than the normal CP treatment by 8 points during d 24 to 42 and by 5 points during d 10 to 42. When GAA spared Arg at 100%, FCR was lower than the normal CP treatment by 5 points during d 24 to 42 and by 3 points during d 10 to 42. FCR increased when betaine was added to low CP + GAA 50 during d 24 to 42 and d 10 to 42 but did not change when it was added to low CP + GAA 100 and low CP + GAA 150 in any phases.

### Carcass Cuts and Internal Organs

The effect of low CP diets with GAA and betaine on absolute and relative (g/kg live weight) organ weights and toe ash of broilers are presented in Table 6. Dietary treatments had significant effects (*P* < 0.001) on absolute and relative breast weights. The birds offered a low CP diet deficient in Arg had 13.1% lower absolute breast weight and 8.5% lower relative breast weight compared to those offered a normal CP diet. When Arg was added back, absolute and relative breast weights increased and relative breast weight became comparable to the normal CP treatment. When GAA spared Arg at 50, 100, and 150% without or with betaine, absolute and relative breast weights were not affected compared to the low CP + Arg treatment but were higher than the low CP − Arg treatment. Betaine did not affect absolute and relative breast weights when it was added to each level of GAA.

**Table 6:** Absolute (g) and relative (g/kg live weight) organ weights and toe ash of broilers offered low crude protein diets with guanidinoacetic acid and betaine on d 42.

Treatment	Breast meat (g)	Breast meat (g/kg)	Leg piece^10^ (g)	Leg piece (g/kg)	Abdominal fat (g/kg)	Liver (g/kg)	Toe ash^11^ (%)
Normal CP^1^	685.3^a^	189.6^a^	747.7^a^	204.2abc	9.2^c^	15.2^c^	11.2
Low CP - Arg^2^	595.6^c^	173.5^d^	706.0^b^	205.8^ab^	12.0^a^	17.5^ab^	11.1
Low CP + Arg^3^	647.7^b^	183.8abc	727.9^ab^	206.5^a^	9.9^bc^	16.6^ab^	11.4
Low CP + GAA 50^4^	649.0^b^	179.1^cd^	739.7^a^	204.1abc	10.2^bc^	16.4^b^	11.4
Low CP + GAA 100^5^	673.5^ab^	187.1^ab^	729.4^ab^	200.6abc	9.8^bc^	16.6^ab^	11.2
Low CP + GAA 150^6^	675.1^ab^	187.0^ab^	722.7^ab^	200.5abc	10.4^bc^	17.7^a^	11.6
Low CP + GAA 50 + betaine^7^	651.1^b^	184.1abc	722.4^ab^	204.2abc	9.7^bc^	16.9^ab^	10.8
Low CP + GAA 100 + betaine^8^	644.9^b^	181.4^bc^	705.4^b^	198.5^c^	10.1^bc^	16.3^b^	11.4
Low CP + GAA 150 + betaine^9^	653.0^b^	183.6abc	709.5^b^	199.5^c^	10.9abc	17.3^ab^	11.2
SEM	4.202	0.087	3.412	0.685	0.164	0.015	0.264
*P* value	< 0.001	< 0.001	< 0.05	< 0.05	< 0.001	< 0.01	0.206

a-d Within each treatment factor, means in the same column with a different superscript differ significantly (*P* < 0.05).

1 Diet 1- Normal crude protein diet.

2 Diet 2- Low crude protein diet deficient in Arginine.

3 Diet 3- Low crude protein diet sufficient in Arginine.

4-9 Diets 4 to 9- low CP diets with GAA where 0.1% added L-Arg was spared by GAA at 50% (GAA inclusion rate- 0.2%), 100% (GAA inclusion rate- 0.1%), and 150% (GAA inclusion rate- 0.067%) with and without 0.1% betaine.

10 Leg piece includes combined weight of thigh and drumstick.

11 Percentage ash of dried toe.

Dietary treatments had significant effects (*P* < 0.05) on absolute and relative weights of leg piece (thigh plus drumstick). The birds offered a low CP diet deficient in Arg had 5.6% lower absolute leg piece weight compared to those offered a normal CP diet. When Arg was added back, absolute leg piece weight increased and became comparable to the normal CP treatment. The deficiency of Arg did not affect the relative weight of the leg piece. When GAA spared Arg at 50, 100, and 150% without betaine, absolute and relative leg piece weights were not affected compared to the low CP + Arg treatment. Betaine did not affect absolute and relative leg piece weights when it was added to each level of GAA.

Dietary treatments had a significant effect (*P* < 0.001) on relative abdominal fat pad weight. The birds offered a low CP diet deficient in Arg had 30.4% higher relative abdominal fat pad weight compared to those offered a normal CP diet. When Arg was added back, relative abdominal fat pad weight decreased and became comparable to the normal CP treatment. When GAA spared Arg at 50, 100, and 150% without or with betaine, relative abdominal fat pad weight was lower than the low CP – Arg treatment and comparable to the low CP + Arg treatment. Betaine did not affect relative abdominal fat pad weight when it was added to each level of GAA.

Dietary treatments had a significant effect (*P* < 0.01) on relative liver weight. The birds offered a low CP diet deficient in Arg or a low CP diet sufficient in Arg had higher relative liver weight compared to those offered a normal CP diet. When GAA spared Arg at 50, 100, and 150% without or with betaine, relative liver weight was not affected compared to the low CP + Arg treatment but was lower than the normal CP treatment. Betaine did not affect relative liver weight when it was added to each level of GAA. Dietary treatments had no effect (*P* > 0.05) on toe ash content.

### Breast Meat Creatine Concentration

**Figure 1:**
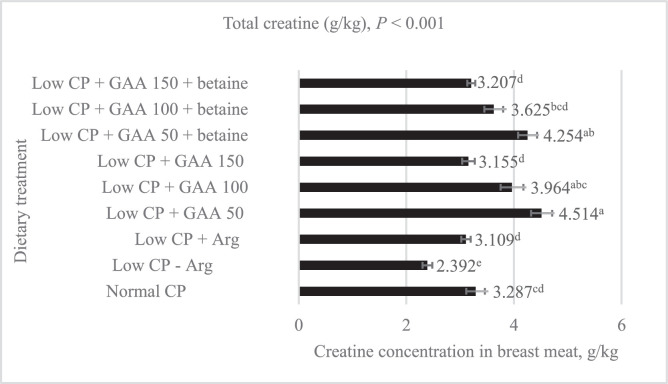
Effect of low crude protein diets with guanidinoacetic acid and betaine on creatine concentration in broiler breast meat at d 42.

The effect of low CP diets with GAA and betaine on breast meat Cr concentration is presented in Figure 1. Dietary treatments had a significant effect (*P* < 0.001) on breast meat Cr concentration. The birds offered a low CP diet deficient in Arg had 27.2% lower Cr concentration in breast meat compared to those offered a normal CP diet. When Arg was added back, breast meat Cr level increased by 30% and was comparable to the normal CP treatment. When GAA spared Arg at 150% without or with betaine, breast meat Cr level was higher than the low CP − Arg treatment and comparable to the low CP + Arg treatment. When GAA spared Arg at 100% without betaine, breast meat Cr level was higher than the low CP + Arg treatment by 27.5% and comparable to the normal CP treatment. When GAA spared Arg at 50% without betaine, breast meat Cr level was higher than the low CP + Arg treatment by 45.2% and the normal CP treatment by 37.3%. Betaine did not affect breast meat Cr concentration when it was added to each level of GAA.

### Meat Quality

The effect of low CP diets with GAA and betaine on broiler breast meat drip loss, pH, moisture, cooking loss, shear force, and color (L*, a*, b*) values are presented in Table 7.

**Table 7:** Meat quality of broilers offered low crude protein diets with guanidinoacetic acid and betaine on d 42.

Treatment	Drip loss, %	Cooking loss, %	pH	Moisture, %	Shear force, N	Breast meat color L* a* b*		
Normal CP^1^	1.45^ab^	21.4^cd^	5.96^b^	74.8bcd	19.55	53.4^c^	4.8	1.9^ab^
Low CP - Arg^2^	1.99^a^	20.6^d^	6.07^a^	74.5^d^	17.14	53.2^c^	5.3	1.3^b^
Low CP + Arg^3^	1.37^b^	21.6bcd	5.95^b^	74.6^cd^	18.19	55.8^b^	4.9	2.2^a^
Low CP + GAA 50^4^	1.41^ab^	23.2^a^	5.80^de^	75.7^a^	19.10	58.2^a^	4.6	2.0^ab^
Low CP + GAA 100^5^	1.36^b^	21.5^cd^	5.88bcd	74.8bcd	21.13	56.0^b^	4.8	2.1^a^
Low CP + GAA 150^6^	1.57^ab^	22.8abc	5.87bcd	75.1abcd	20.11	56.3^ab^	4.9	2.7^a^
Low CP + GAA 50 + betaine^7^	1.54^ab^	23.1^a^	5.79^e^	75.4^ab^	21.12	58.3^a^	4.7	2.0^ab^
Low CP + GAA 100 + betaine^8^	1.35^b^	22.2abc	5.86bcd	75.0bcd	20.99	56.8^ab^	4.8	2.6^a^
Low CP + GAA 150 + betaine^9^	1.61^ab^	22.2abc	5.90^bc^	75.3abc	19.26	56.0^b^	5.0	2.5^a^
SEM	0.127	0.485	0.029	0.209	1.528	0.688	0.232	0.251
*P* value	< 0.05	< 0.01	< 0.001	< 0.01	0.588	< 0.001	0.594	< 0.01

a-e Within each treatment factor, means in the same column with a different superscript differ significantly (*P* < 0.05).

1 Diet 1- Normal crude protein diet.

2 Diet 2- Low crude protein diet deficient in Arginine.

3 Diet 3- Low crude protein diet sufficient in Arginine.

4-9 Diets 4 to 9- low CP diets with GAA where 0.1% added L-Arg was spared by GAA at 50% (GAA inclusion rate- 0.2%), 100% (GAA inclusion rate- 0.1%), and 150% (GAA inclusion rate- 0.067%) with and without 0.1% betaine.

### Drip Loss

Dietary treatments led to a significant difference (*P* < 0.05) in breast meat drip loss. The birds offered a low CP diet sufficient in Arg had lower breast meat drip loss compared to those offered a low CP diet deficient in Arg. When GAA spared Arg at 50, 100, and 150% without or with betaine, drip loss was not affected compared to the low CP + Arg treatment. Betaine did not affect drip loss when it was added to each level of GAA.

### pH

Dietary treatments had a significant effect (*P* < 0.001) on breast meat pH. The birds offered a low CP diet deficient in Arg had higher breast meat pH compared to those offered a normal CP diet. When Arg was added back, pH decreased and became similar to the normal CP treatment. When GAA spared Arg at 50% without or with betaine, breast meat pH decreased compared to the Arg deficient and Arg sufficient low CP and normal CP treatments. When GAA spared Arg at 100 and 150% without or with betaine, breast meat pH was not affected compared to the low CP + Arg treatment. Betaine did not affect breast meat pH when it was added to each level of GAA. As shown in Table 8, the breast meat pH was negatively correlated with breast meat Cr concentration (r = −0.49, *P* < 0.001).

**Table 8:** Pearson correlation coefficient (r) between breast meat creatine concentration and meat quality parameters.

Parameter	Breast meat moisture	Breast meat pH	Relative weight of abdominal fat	FCR (d 10-42)
Breast meat creatine concentration	0.33	−0.49	−0.37	−0.70
*P*-value	< 0.01	< 0.001	< 0.01	< 0.001

### Moisture

Dietary treatments had a significant effect (*P* < 0.01) on the moisture content of breast meat. The birds offered a low CP diet deficient in Arg or a low CP diet sufficient in Arg did not affect breast meat moisture compared to those offered a normal CP diet. When GAA spared Arg at 50% without or with betaine, breast meat moisture increased compared to the low CP + Arg and low CP – Arg treatments. When GAA spared Arg at 100 and 150% without or with betaine, breast meat moisture was not affected compared to the low CP + Arg treatment. Betaine did not affect breast meat moisture when it was added to each level of GAA. As shown in Table 8, the moisture content in breast meat was positively correlated with breast meat Cr concentration (r = 0.33, *P* < 0.01).

### Shear Force

Dietary treatments had no effect (*P* > 0.05) on the shear force of breast meat.

### Cooking Loss

Dietary treatments had a significant effect (*P* < 0.01) on cooking loss of breast meat. The birds offered a low CP diet deficient in Arg or a low CP diet sufficient in Arg did not affect cooking loss compared to those offered a normal CP diet. When GAA spared Arg at 50% without or with betaine, cooking loss increased compared to the low CP + Arg, low CP – Arg and normal CP treatments. When GAA spared Arg at 100 and 150% without or with betaine, the cooking loss was not affected compared to the low CP + Arg treatment. Betaine did not affect cooking loss when it was added to each level of GAA.

### Color

Dietary treatments led to significant difference in breast meat color values for lightness (L *, *P* < 0.001) and yellowness (b *, *P* < 0.01) but not redness (a *, *P* > 0.05). The birds offered a low CP diet deficient in Arg did not affect breast meat L * and b * values compared to those offered a normal CP diet. When Arg was added back, L * value increased and became higher than the low CP – Arg and normal CP treatments whereas b * value increased and became similar to the normal CP treatment. When GAA spared Arg at 50% without or with betaine, breast meat L * values increased compared to the low CP – Arg, low CP + Arg and normal CP treatments. When GAA spared Arg at 100% and 150% without or with betaine, breast meat L * values were similar to the low CP + Arg treatment but higher than normal CP treatment. When GAA spared Arg at 50%, 100%, and 150% without or with betaine, b * value was not affected compared to the low CP + Arg treatment. Betaine did not affect breast meat L * and b * values when it was added to each level of GAA.

### Correlation Between Breast Meat Cr Concentration and Performance Parameters

**Figure 2:**
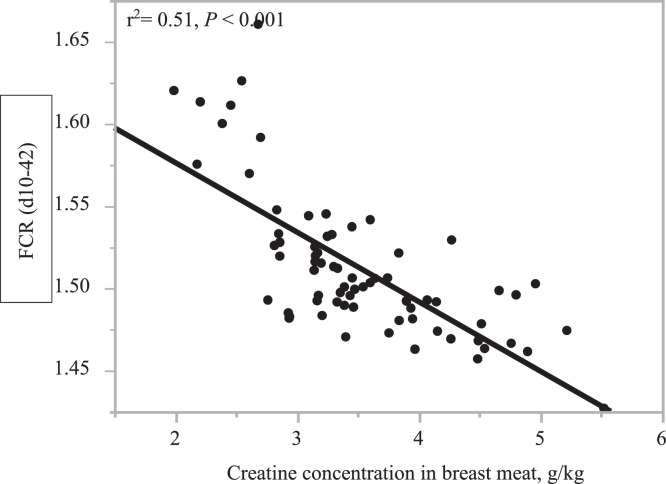
Linear relationship between breast meat creatine concentration and FCR (d 10–42) of broilers offered low crude protein diets with guanidinoacetic acid and betaine.

**Figure 3:**
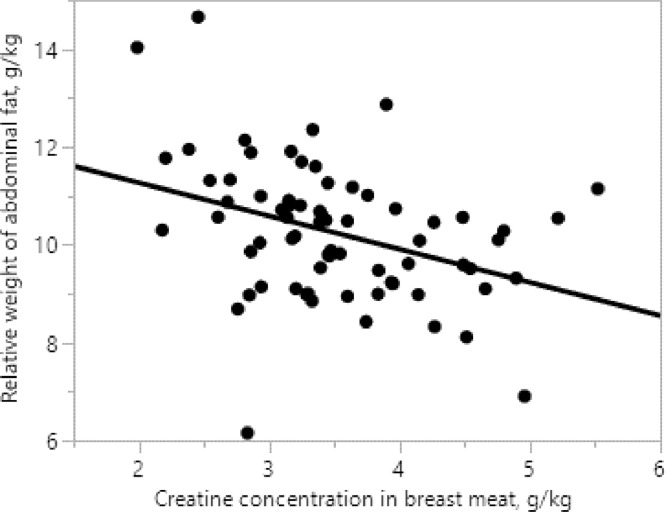
Inverse linear relationship between breast meat creatine concentration and relative weight of abdominal fat at d 42 of broilers offered low crude protein diets with guanidinoacetic acid and betaine (*P* < 0.01).

As shown in Table 8, breast meat Cr concentration was negatively correlated to FCR (r = −0.70, *P* < 0.001) and relative weight of abdominal fat (r = −0.37, *P* < 0.01). The linear regression between FCR (d 10–42) and breast meat Cr concentration was FCR = 1.6612 − 0.0423 × Cr, g/kg (r^2^ = 0.51, *P* < 0.001) as shown in Figure 2. There was an inverse linear relationship (*P* < 0.01) between breast meat Cr concentration and relative weight of abdominal fat pad as shown in Figure 3.

### White Striping

**Figure 4:**
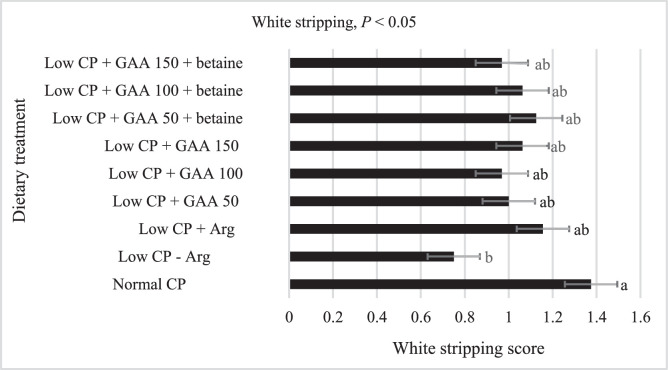
Effect of low crude protein diets with guanidinoacetic acid and betaine on white stripping score in broiler breast meat at d 42.

The effect of low CP diets with GAA and betaine on white striping scores in breast meat of broilers is presented in Figure 4. Dietary treatments had a significant effect (*P* < 0.05) on white striping scores in breast meat. The birds offered a low CP diet deficient in Arg had a lower white striping score in breast meat compared to those offered a normal CP diet. When Arg was added back, the white striping score increased and became similar to the birds offered a normal CP diet. When GAA spared Arg at 50, 100, and 150% without or with betaine, white striping scores in breast meat were not affected compared to the birds offered low CP diets sufficient in Arg. Betaine did not affect white striping scores when it was added to each level of GAA.

### Wooden Breast

**Figure 5:**
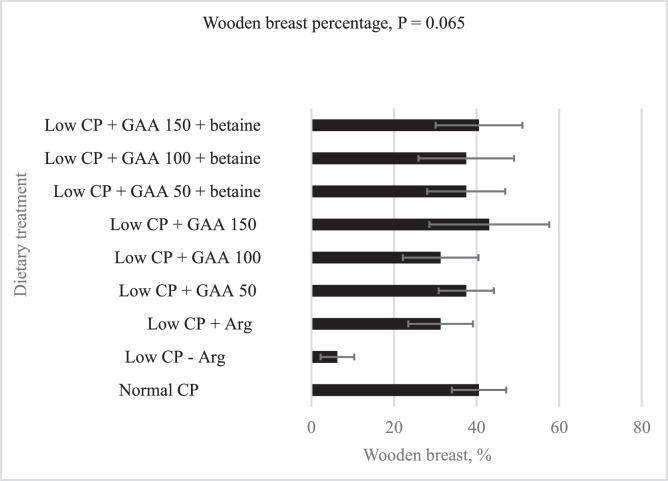
Effect of low crude protein diets with guanidinoacetic acid and betaine on wooden breast incidence in broilers at d 42.

The effect of low CP diets with GAA and betaine on wooden breast incidence of broilers is presented in Figure 5. Dietary treatments tended (*P* = 0.065) to affect wooden breast incidence with the lowest percentage being observed in the birds offered a low CP diet deficient in Arg.

## DISCUSSION

In a wheat-sorghum based low CP diet, Arg concentration is naturally low which makes Arg a costly nutrient and contributes significantly to feed cost. Dietary GAA may be used as a partial replacement to supplemental Arg as it reduces the requirement of Arg for Cr synthesis. Adding GAA produces more Cr in the muscle cells than adding a higher level of L-Arg as the latter may be metabolized quickly in the blood and may not be the most efficient way of maximizing Cr. A sufficient level of dietary methyl donor is required to convert GAA to Cr, especially when the dietary level of GAA is higher, to prevent homocysteine toxicity (Ostojic et al., 2013). Thus, it is of interest to study the rate at which GAA may replace supplemental L-Arg in moderately low CP diets with and without a methyl donor to maximize growth performance, breast meat yield, and Cr synthesis.

This study demonstrated the magnitude of Arg deficiency on growth performance, breast meat yield and meat quality of broilers offered a moderately low CP diet. During 10 to 42 d, the birds offered 15 g/kg low CP Arg deficient diet had 7.8% lower weight gain, 10 points higher FCR, 8.5% lower breast meat yield, 30.4% more abdominal fat pad and 27.2% lower Cr concentration in breast meat compared to those offered a normal CP diet. Some of these observations are in line with the recent findings (Dilger et al., 2013; Castro et al., 2019; Dao et al., 2021) and highlight the negative effects of Arg deficiency in broilers. Arginine deficiency also increased breast meat pH which may favor microorganism growth and decrease the shelf life of breast meat. Arginine is an essential amino acid for chickens as they lack a functional urea cycle and are thus unable to synthesize it endogenously Khajali and Wideman, 2010. Any deficiency in dietary Arg will affect body protein synthesis as other amino acids that are needed for protein accretion are not effectively used at the site of protein synthesis and the excess is deaminated. The energy required for muscle protein synthesis is higher than that required for protein deamination and thus this excess energy may get deposited as fat resulting in a higher abdominal fat pad observed in birds offered Arg deficient diets in this study. A low incidence of woody breast and white striping scores in birds offered Arg deficient diet compared to those offered Arg sufficient and normal protein diets may be attributed to a slower growth rate in those birds. Studies have reported a positive correlation between growth rate and woody breast or white striping issues with reduced growth rate resulting in low occurrence and severity of white striping and wooden breast issues (Lorenzi et al., 2014; Cruz et al., 2017; Kawasaki et al., 2018) which may explain the phenomena observed in breast meat. The efficacy of adding Arg back to the Arg deficient low CP diet on growth performance was also demonstrated in this study. When Arg was added back to the Arg deficient low CP diet, weight gain, feed efficiency, breast meat yield, and breast meat Cr concentration increased, abdominal fat pad decreased and became comparable to the normal CP treatment. Some of these results are in line with the recent findings (Dilger et al., 2013; Dao et al., 2021) and highlight the importance of supplemental Arg in a low CP diet fed to broilers as described above. Although the addition of Arg to the Arg deficient low CP diet had no significant effect on wooden breast and white striping issues, Bodle et al. (2018) reported a lower wooden breast issue in broilers fed diets with higher dietary dArg to dLys ratio compared to those fed an Arg sufficient diet. This was thought to be related to the vasodilation effect of nitric oxide which is produced from L-Arg by the enzyme nitric oxide synthase Khajali and Wideman, 2010 that enhance blood flow to the breast muscle and alleviate the hypoxic condition usually observed in breasts affected by these issues (Boerboom et al., 2018). Considering the vasodilation effect of Arg, it is plausible to think that a higher dietary dArg to dLys ratio beyond that used in the current study would reduce breast meat myopathies in broilers fed low CP diets. The addition of Arg to the Arg deficient RP diet decreased breast meat drip loss and pH and increased color to make them comparable to the normal CP treatment. This further highlights the importance of dietary Arg to improve the shelf life of breast meat by lowering pH and drip loss and enhancing color.

The important research question of this study was whether the benefits of supplemental Arg in Arg deficient low CP diets could be maintained or further enhanced by partly replacing it with GAA? When GAA is used to spare Arg in broiler diets, Arg can be diverted for other metabolic functions such as muscle accretion, cell signaling, and hormone release rather than Cr formation (Portocarero and Braun, 2021) for which GAA is more efficient and economic. In this study, 1 kg/MT of added L-Arg in low CP diet was replaced with GAA at replacement rates of 50 (2.0 kg/MT), 100 (1.0 kg/MT), and 150% (0.067 kg/MT) with or without betaine. The hypothesis was that GAA could spare Arg at 150% to maintain growth performance and meat quality benefits and this would be further enhanced at higher inclusion rates or higher replacement rates of 100 and 50%. Guanidinoacetic acid together with betaine was thought to improve growth performance and breast muscle Cr concentrations further as betaine would act as a methyl donor providing methyl group to GAA for Cr synthesis. The growth performance, carcass yield and meat quality data in this study supported our claim that GAA could spare Arg at 150% in low CP diets. For example, when GAA spared Arg at 150%, feed intake, weight gain, FCR, breast meat, leg piece, abdominal fat, breast meat moisture, drip loss, pH, color, breast meat Cr concentration, and white stripping score were similar to the low CP + Arg and normal CP treatments. It should be noted that when L-Arg or GAA was added to the Arg deficient low CP diet, the magnitude of improvement in breast meat weight was higher with GAA 150% (13.3% increment) than with Arg (8.7% increment). When this is converted to an absolute scale, low CP + GAA 150% treatment provided 27 g more breast meat than low CP + Arg treatment. Literature findings suggest that the Arg-sparing effect of GAA may be between 75 and 142% with a theoretical value up to 149% (Michiels et al., 2012; Dilger et al., 2013; De Groote et al., 2018; Lemme et al., 2018; Khajali et al., 2020) and the range was reported depending on the growth performance parameters investigated. Our results showed that 1 kg/MT of added L-Arg can be spared by GAA at 150% in moderately low CP diets without affecting growth performance and an additional payback through 27 g increased breast meat yield. Therefore, GAA may allow the use of moderately reduced CP diets in the industry to maximize profitability.

Interestingly, there was a variable response in FCR with different levels of GAA in a low CP diet. The higher levels of GAA decreased FCR beyond that produced by the normal CP treatment. When GAA spared Arg at 100%, FCR was similar to low CP + Arg treatment but 3 points lower than the normal CP treatment. When GAA spared Arg at 50%, FCR was 5 points lower than the low CP + Arg and normal CP treatments. The linear improvements in feed efficiency of broilers with increasing levels of GAA (from 0.067 to 0.2%) in a low CP diet indicates that more Arg was spared from serving as a precursor for Cr synthesis and was available for other metabolic functions, primarily lean tissue accretion (DeGroot et al., 2018). This was evident from the linear relationship between breast meat Cr concentration and FCR observed in our study with high Cr concentrations resulting in decreased FCR (Figure 2). These results corroborate previous observations of improved FCR with higher levels of dietary Arg. For example, Barekatain et al., (2019) reported improved FCR with a dietary Arg level of approximately 140% of the Ross 308 recommendations. Similarly, Corzo, (2012) reported dArg to dLys optimal ratios of 106% for feed intake, 108% for weight gain, and 114% for FCR in Ross 708 broilers from 1 to 18 d of age. More recently, Corzo et al., (2021) reported an optimum ratio of 105 and 108% for weight gain and FCR, respectively from 1 to 25 days of age. It is evident from the above studies that the requirement of dArg to dLys ratio increases with age and is higher for FCR than for weight gain. In a low CP feeding context, the requirement of dArg to dLys ratio for maximizing the growth performance of broilers is not clear. If we translate the findings from the above studies, more supplemental Arg will be required in low CP diets to meet higher dArg to dLys ratios. In general, free Arg is cleared quickly in the blood and is not the most efficient way of maximizing Cr. In our study, the addition of Arg or GAA in Arg deficient low CP diet restored the Cr availability in breast meat and improved growth performance but there was a linear increase in breast meat Cr concentration with increasing levels of GAA and this translated to improved FCR. In low CP diets, it may be efficient to partially replace added Arg with GAA for maximizing Cr in muscles. This will increase energy availability and possibly metabolic utilization of nutrients for growth resulting in improved FCR. To what extent added L-Arg can be replaced with GAA at higher dArg to dLys ratios in low CP diets should be explored in the future. A meta-analysis by Khajali et al. (2020) using 32 published papers on GAA found that the most consistent effect of GAA supplementation in broilers was on FCR. The improvements in FCR were dose-dependent and in the range of 4.5 to 8.8 points at dietary GAA inclusions between 0.06 and 0.12% which is close to the improvements observed in our study. However, at 50% Arg replacement, pH of breast meat decreased, moisture, cooking loss and lightness (L*) increased compared to low CP + Arg and normal CP treatments. A lower pH, higher cooking loss and higher lightness (L*) and yellowness (b*) were also observed in breast meat as a result of GAA supplementation in Arg deficient diet in a previous study (Michiels et al., 2012). However, in both the studies, the effects were small and may not have implications in the retail value of breast meat but it does indicate that Arg or GAA supplementation will decrease breast meat pH, make meat color better, and possibly reduce microbial growth and increase the shelf life of meat.

In this study, the analyzed Cr concentrations in the diets were below the minimum reporting limit set in the assay (i.e., 20 mg/kg) which confirms that both the normal and the low CP diets contained negligible amount of Cr which is normally expected in diets exclusively based on grains and vegetable protein meals (Khajali et al., 2020). Betaine did not increase breast meat Cr concentration at any levels of GAA. There are 2 possible reasons for the lack of betaine response in low CP diets with GAA. First, the low CP diet already contained adequate levels of methyl donors (e.g., methionine and choline) and there was no need for additional methyl donors to convert dietary GAA into Cr. Second, the maximum level of GAA that was used in this study (i.e., 0.20%) was not high enough to saturate muscle tissues with Cr and to instigate any negative effects on performance as seen in the previous study (Dao et al., 2021). Further research is warranted to determine the maximum level of GAA that can be used to spare Arg in low CP diets and the role of betaine in such cases should be explored. The efficiency at which Arg and GAA may deposit Cr in breast meat was demonstrated in this study. For example, when Arg was added back to the Arg deficient diet, breast meat Cr concentration increased by 30% and when this added Arg was partly replaced with GAA at 150, 100, and 50%, breast meat Cr concentrations increased by 31.9, 65.7, and 88.7%, respectively compared to that provided by the Arg deficient diet. This demonstrates that dietary GAA is a more potent Cr source than Arg. The positive correlation between breast meat total Cr concentration and breast meat moisture observed in this study highlights the muscle Cr/phosphocreatine role to draw water into the muscle cells and possibly result in increased breast meat weight. The negative correlation between breast meat Cr concentration and relative weight of abdominal fat suggests that Cr improves energy efficiency and increases fat oxidation.

## CONCLUSIONS

Guanidinoacetic acid can be used to replace 150% of Arg in moderately low CP diets offered to broilers for comparable growth performance, meat quality and conversion or production of Cr. For enhanced feed efficiency and higher muscle Cr deposition, higher replacement rates may be considered. Increased FCR and abdominal fat pad weight are major issues in broilers fed low CP diets but a partial replacement of dietary Arg with GAA may help to solve these issues. Using GAA by the method described herein may be a nutritional strategy for the successful implementation of a low CP feeding program for broilers.
